# Probing the long-lived photo-generated charge carriers in transition metal dichalcogenides by time-resolved microwave photoconductivity

**DOI:** 10.1515/nanoph-2021-0741

**Published:** 2022-02-24

**Authors:** Artur P. Herman, Szymon J. Zelewski, Kamil Misztal, Robert Kudrawiec

**Affiliations:** Department of Semiconductor Materials Engineering, Wroclaw University of Science and Technology, Wybrzeże Wyspiańskiego 27, Wrocław, 50-370, Poland

**Keywords:** charge carrier lifetime, exciton dissociation, time-resolved microwave photoconductivity, transition metal dichalcogenides

## Abstract

Understanding the dissociation of excitons into long-lived free charge carriers is a crucial issue when considering the applications of transition metal dichalcogenides (excitonic semiconductors) oriented toward the use of solar energy (such as photovoltaics or photocatalysis). In our work, long-lived carriers have been observed by time-resolved microwave photoconductivity (TRMC) for the first time in both atomically thin and bulk MoS_2_, MoSe_2_, WS_2_, and WSe_2_ crystals. The lifetime of majority carriers is close to microseconds and can even reach several microseconds due to different contribution of surface and defect states, as well as surface band bending (bulk). The three components depend on the material and vary from sample to sample, therefore determining the dynamics of the TRMC signal. The rise time of TRMC signal was found to be in the range of 0.1–0.2 μs and as it depends on the studied material it can be speculated that it is related to the dissociation time of excitons captured by traps.

## 1 Introduction

Transition metal dichalcogenides (TMDs) such as MX_2_ (M = Mo, W; X = S, Se) belong to the exciting class of layered materials of unprecedented physiochemical properties. The latter make them perfect candidates for applications in sustainable technologies of tomorrow such as *inter alia* (opto)electronics (particularly photovoltaics) and solar-driven chemistry (artificial photosynthesis, photocatalysis) [1], [2], [3], [4], [5], [6], [7]. When a bulk TMD is thinned down to a monolayer, both its electronic and optical properties change [8], [9], [10], [11], [12]. The transition from bulk to monolayer form should clearly affect the carrier dynamics as the change in the electronic band structure is accompanied by the phenomena related to the enhanced role of surface in atomically thin layers. So far, the carrier dynamics in monolayer TMDs has been the subject of many reports employing such methods as time-resolved photoluminescence (TRPL), pump-probe techniques, and time-resolved terahertz spectroscopy [13], [14], [15], [16], [17], [18], [19], [20], [21], [22], [23], [24], [25], [26], [27], [28], [29], [30], [31], [32], [33], [34]. In the case of their bulk counterparts, the issue of charge carrier dynamics has not been studied comprehensively [35] most likely due to the lack of photoluminescence.

TRPL studies of atomically thin MX_2_ showed very fast carrier dynamics [13, 15, 16, 28], [29], [30], [31, 34]. It was observed that the decay of PL related to the emission of free excitons ranges from a few to several hundred ps [13, 15, 16, 18, 28], [29], [30], [31, 34]. A longer PL decay time was observed for localized excitons [29, 30, 36], [37], [38] but this dynamics is still much faster than ns in many cases [29, 30] and approaching 200 ns for electron-beam irradiated WSe_2_ monolayers [36]. The expected intrinsic carrier/exciton lifetime may be even longer than ns [20, 33, 39, 40], but non-radiative recombination processes reduce the PL decay time compromising the ability to determine the intrinsic lifetime. It is obvious, and generally accepted, that the majority of non-radiative recombination processes in atomically thin MX_2_ involve the surface. The encapsulation of MX_2_ layers with *h*-BN allows to reduce the impact of non-radiative recombination and hence to extend the PL decay time [22, 24]. However, the detected PL decay times for such samples are still very short. An elongation of the PL decay time to a few ns was observed for MX_2_ monolayers treated with oleic acid ligands [40, 41]. Ultra-fast carrier dynamics in TMDs was also observed using pump-probe techniques [14, 19, 25, 32] and time-resolved THz spectroscopy [17, 23, 42, 43]. Pump-probe techniques, unlike PL, allow to probe non-radiative states, such as indirect excitons in *k*-space which should have a much longer lifetime, though even in this case the reported lifetimes were below ns [25, 27]. Indirect excitons were also observed using TRPL for bi- and tri-layer WS_2_. The PL decay time for such excitons was longer than for exciton emission from the WS_2_ monolayer and approached ns [34]. In this case, the defect states as well as the surface also influenced the PL dynamics, but it is not easy to eliminate the non-radiative processes and determine the intrinsic exciton lifetime as the carrier dynamics in MX_2_ is a tough nut to crack. A better understanding of the carrier dynamics in TMDs requires further research using other techniques that are complementary to TRPL. One of them is the time-resolved microwave (photo)conductivity (TRMC) [44], [45], [46], [47], [48], [49], [50].

Quite surprisingly, TRMC has never been used in systematic studies of charge carrier dynamics in MX_2_. There is indeed literally one report devoted to TRMC studies of MoS_2_ and MoSe_2_ powders [51], but there are no TRMC studies focused on monolayer and bulk MX_2_. TRMC is a contactless method and consists of stimulating the sample with a short pulse of light (usually a laser pulse) that generates carriers in the conduction band (and/or the valence band). The sample is simultaneously irradiated using microwaves and the changes in the microwave reflection intensity (interpreted as changes in sample’s conductivity) are measured as a function of time [44], [45], [46], [47], [48], [49], [50]. Carrier concentration variation is described by an exponential decay with a characteristic constant, which is understood as the carriers’ lifetime. It is worth emphasizing here that in order to observe TRMC signal the presence of only one type of carriers (electrons or holes) is sufficient, overcoming the fundamental limitation of TRPL where both of them must meet and recombine to generate emission.

In the context of TMDs, it should be noted that due to high exciton binding energy the excitonic effects dominate over free-carrier emission in optical spectroscopy even at high temperatures when the thermal energy is high [22], while TRMC is an exciton insensitive method as it probes changes in conductivity. This may be one of the reasons why this method has not yet been applied to study TMDs. One can speculate that the second reason is limited ability to detect short lifetimes. Current TRMC systems use lasers with a pulse width of a few nanoseconds and oscilloscopes operating at frequencies up to 1 GHz. Using such equipment allows to determine carrier lifetimes longer that ∼10 ns, [52], while the carrier dynamics reported so far for atomically thin TMDs is much shorter. For bulk TMDs the TRMC method seems to be a proper tool to study the carrier dynamics as for Si or SiC [44], [45], [46], [47], [48], and therefore it is interesting to apply this method to study bulk TMDs both as crystals themselves and as a reference for understanding the carrier dynamics in atomically thin TMDs.

Herein, we used TRMC to study both atomically thin and bulk MoS_2_, MoSe_2_, WS_2_, and WSe_2_ crystals and observed TRMC signal indicating long-lived carriers in these materials. The mechanism leading to the prolonged carrier lifetime in TMDs is proposed and discussed within this work. Additionally, the rise time of the TRMC signal is discussed in terms of exciton dissociation time.

## 2 Results and discussion

**Figure 1:**
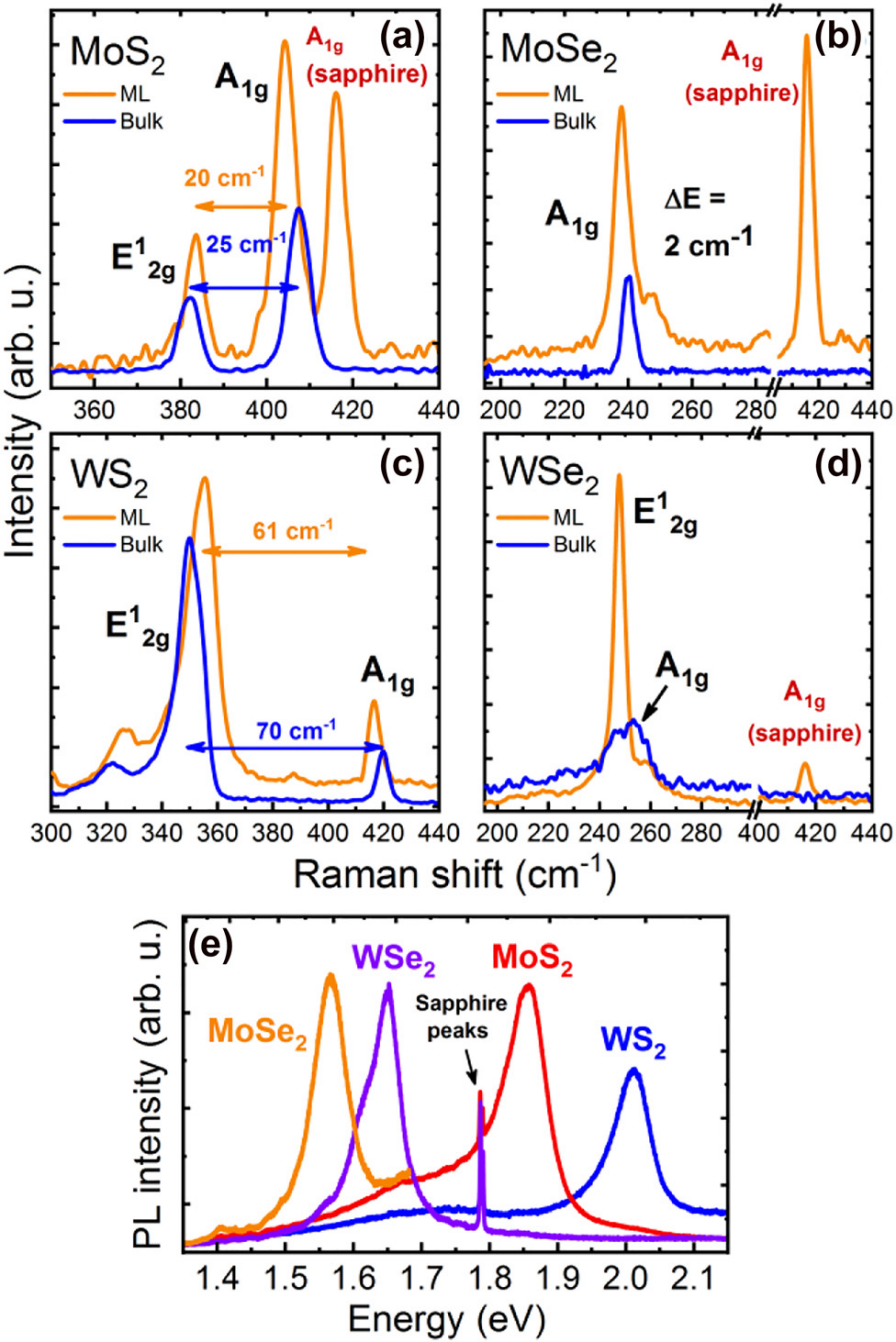
Comparison of room temperature Raman scattering of monolayer (ML) and bulk TMDs (a)–(d) and photoluminescence spectra of ML TMDs (e). Sharp emission peaks at 694 nm (∼1.79 eV) originate from the sapphire substrate, common for all MLs.

TRMC measurements were performed at room temperature under the same excitation conditions for monolayer and bulk samples of MoS_2_, MoSe_2_, WS_2_, and WSe_2_. Micro-Raman and photoluminescence (PL) characteristics for these samples are shown in Figure 1. Panels (a)–(d) compare Raman scattering spectra for monolayer and bulk TMDs. The spectral positions of Raman peaks in bulk TMDs are identified according to previous studies [11, 53], [54], [55] and their shifts for monolayer samples are attributed to changes in intra- and interlayer coupling, which are associated with the reduction of crystal thickness down to a monolayer. The recorded Raman spectra uniquely identify the studied samples as good quality bulk and atomically thin TMDs. Moreover, the observation of strong PL peaks in Figure 1(e) proves that the studied samples are atomically thin, as the exciton emission at these energies is characteristic only for monolayer TMDs [8, 9, 11, 12, 21]. PL was not observed for bulk TMDs due to indirect band gap character.

**Figure 2:**
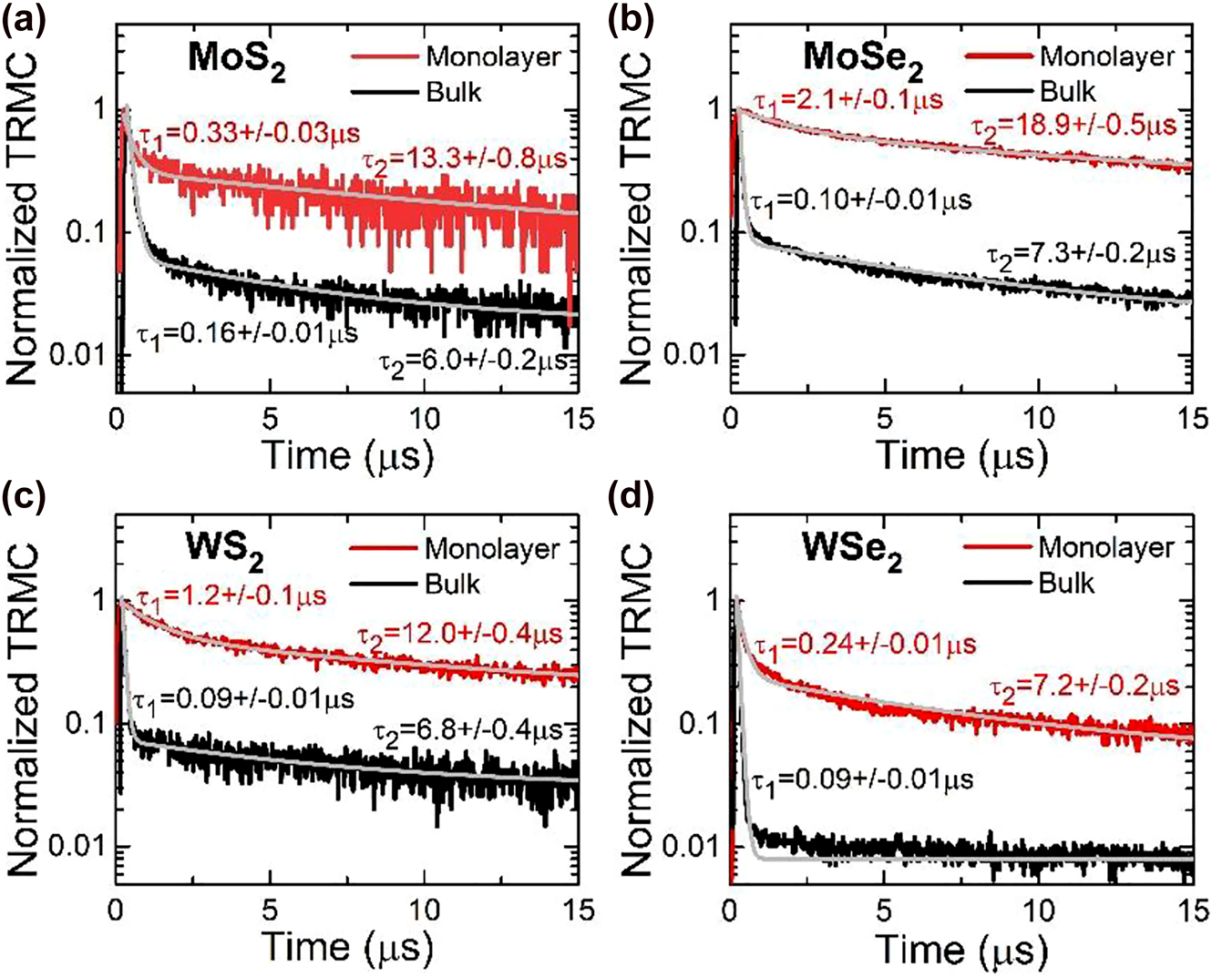
Normalized transient signal of monolayer (red curves) and bulk (black curves) TMDs obtained at room temperature. The gray lines represent the fits with bi-exponential decay.

A direct comparison of the normalized TRMC transients for the four materials is shown in Figure 2. In order to extract the decay time from TRMC measurements, the decay curves have been fitted by a sum of two exponential decays: $I\left(t\right)=I_{1}e^{-t/\tau 1}+I_{2}e^{-t/\tau 2}+y$ , where *I*_1_ and *I*_2_ is the TRMC signal intensity for the process (1) and (2) at the fit beginning, *t* is the time, *y* is the offset, which appears due to a constant signal on the detector and is without physical interpretation in this analysis, and *τ*_1_ and *τ*_2_ is the decay time for the process (1) and (2), which describes the carrier dynamics in the investigated sample.

For monolayer samples (red curves) a long living component can be clearly observed. In addition, a component with shorter decay times can be identified for these samples, especially for WSe_2_. TRMC transients for the four monolayer samples have been fitted by bi-exponential decay and the determined decay times are given in Figure 2. These long-term decays are characterized by times in the order of several microseconds, while the short-term decays are characterized by times in the order of tenths of a microsecond. The component with the shorter time can be observed for all bulk samples (black curves in Figure 2), whereas the long-term decay is clearly distinguished, with the exception of WSe_2_. The curve in Figure 2(d) shows that the short-term decay of bulk WSe_2_ is dominant and reproduces the temporal signal intensity dependence down to the noise level of the detection system.

**Figure 3:**
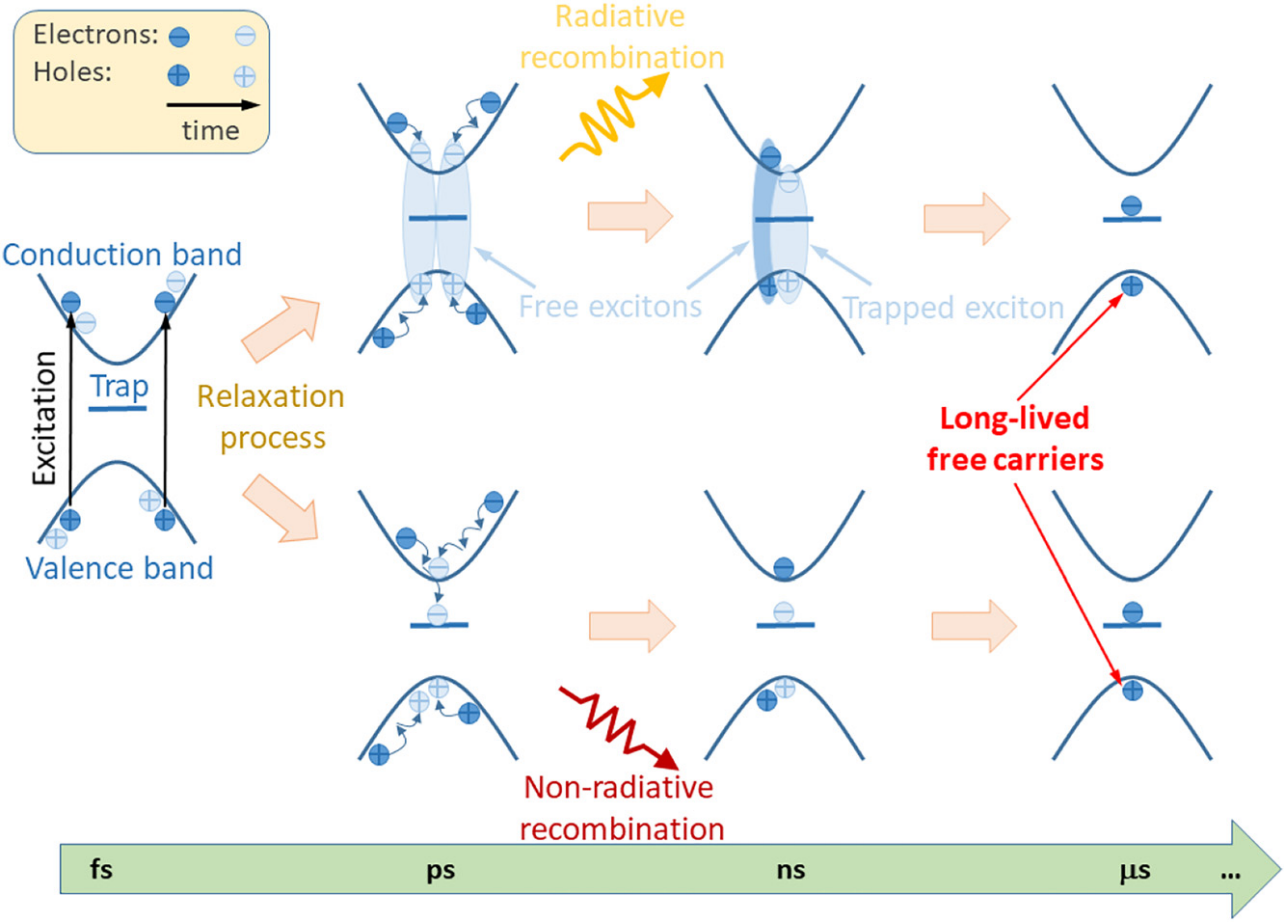
Sketch of carrier dynamics in monolayer TMDs. The trap is a surface state or a state that is associated with a point defect. In general, it can be either an electron trap or a hole trap (the case with an electron trap is shown). Long-lived holes remain in TMD samples due to the rapid capture of electrons (excitons) by electron traps.

The observation of the TRMC signal means that long-lived carriers (electrons or holes) exist in both monolayers and bulk crystals. The origin of these carriers and two components in TRMC can be understood by comprehensive analysis of the carrier dynamics, which is outlined in Figure 3. In this sketch, a trap state is introduced into the forbidden band gap. Such states are typical for semiconductors and have also been proposed to explain the carrier dynamics in TMDs [32, 56]. In general, the origin of this trap state can be diverse and include point defects and/or surface defects/states. Such states have different energies and different occupation time by carriers. The occupation time of surface states may differ significantly from the occupation time of defect states in bulk. In Figure 3, such states are symbolically represented by one state that is located in the forbidden energy gap.

The excitation at 532 nm (2.33 eV) corresponds to energy higher than band gaps of the studied materials (∼1–2 eV), so the photo-generated carriers have a significant excess of energy. The carrier–carrier scattering, which is a very fast process (fs-time scale), thermalizes the carrier energy distribution without reducing the energy in the electron system [57]. The excess carrier energy is transferred to the cold lattice by the electron–phonon scattering with a typical time constant of sub-picoseconds [57], [58], [59], [60]. This process scatters the carriers’ momenta to their respective extrema in the conduction and valence bands [61], [62], [63], [64], creating direct excitons in monolayers and indirect excitons in bulk crystals. Their lifetime is significantly shortened by the non-radiative recombination. Since no PL signal is detected above ns in many TRPL experiments [13, 15, 16, 28], [29], [30], [31, 34], and no PL was observed for the studied samples in TRPL in this time regime, the timescale of radiative recombination of free excitons and their capture is shorter than the laser pulse in our experiment, which is 3–5 ns. For high quality samples (mostly samples with the protected surface) PL can be observed at a few ns [20, 33, 39], [40], [41, 65] but it is still comparable with the width of laser pulse in TRMC experiment and it indicates that the radiative and non-radiative processes in TMDs are very fast [66]. The mechanisms behind non-radiative exciton recombination may be different and may include exciton trapping by the defect/surface followed by its dissociation. One of the important driving forces of exciton dissociation is thermal energy, which cannot be neglected at room temperature, even if the exciton binding energy is a few times higher than kT at room temperature. As a result of exciton dissociation, one carrier may occupy the defect state and then recombine non-radiatively, while the other carrier may remain free in the conduction (valence) band. It can be the long-lived carrier, which is the source of TRMC signal observed in this work for bulk TMDs and their monolayer counterparts. This scenario is illustrated at the top of Figure 3. In this case, we are dealing with a defect-assisted process of exciton dissociation.

Another scenario leading to the same situation is the capture of free carriers by defect/surface states. This situation is illustrated at the bottom of Figure 3. During the electron–phonon relaxation, the geminate electron and hole may also separate in space, creating free carriers rather than excitons. Some of these carriers can be captured by point defects and surface defects/imperfections. The captured carriers, like excitons, do not take part in conductivity but the remaining carriers do not create excitons and can be the source of the TRMC signal. Another evidence of the existence of free carriers in the studied materials are photocurrent measurements [64, 67], [68], [69] and spatiotemporal microwave imaging [70]. However, due to the high trion binding energy in monolayer TMDs [71, 72], the participation of trions in photocurrent cannot be excluded at room temperature. On the other hand, trions show a negative photoconductivity and their dynamics is shorter than ns [29, 73, 74] and therefore the observed TRMC signal cannot be assigned to trions and is an evidence of long-lived carriers in the studied materials.

The lifetime of long-lived carriers is determined by both point defects characteristic for bulk material as well as the surface. The occupation time of surface states is usually longer than the occupation time of traps related to point defects. Therefore, at least two components can be identified in TRMC signal for monolayers and bulk TMDs. The contribution of the two components varies between samples and is significantly different for monolayers. In general, for monolayer TMDs the contribution of bulk-like defect states (e.g. M vacancies in MX_2_) is less important than the contribution of surface defects in contrast to bulk samples. Hence, the dynamics of the TRMC signal observed for monolayers is determined by the dynamics of surface states, while for bulk TMD it results from the occupation times of point defects in bulk. Surface states in bulk TMD also influence carrier dynamics, but their contribution is less significant as the surface/volume ratio (i.e., volume of light penetration in TRMC measurements) is much lower for bulk TMDs than for monolayers. The comparison of TRMC traces for monolayer-bulk TMD pairs allows for identification of components in these traces, which are affected by surface and point defects, i.e. the component with longer time is observed due to surface states.

**Figure 4:**
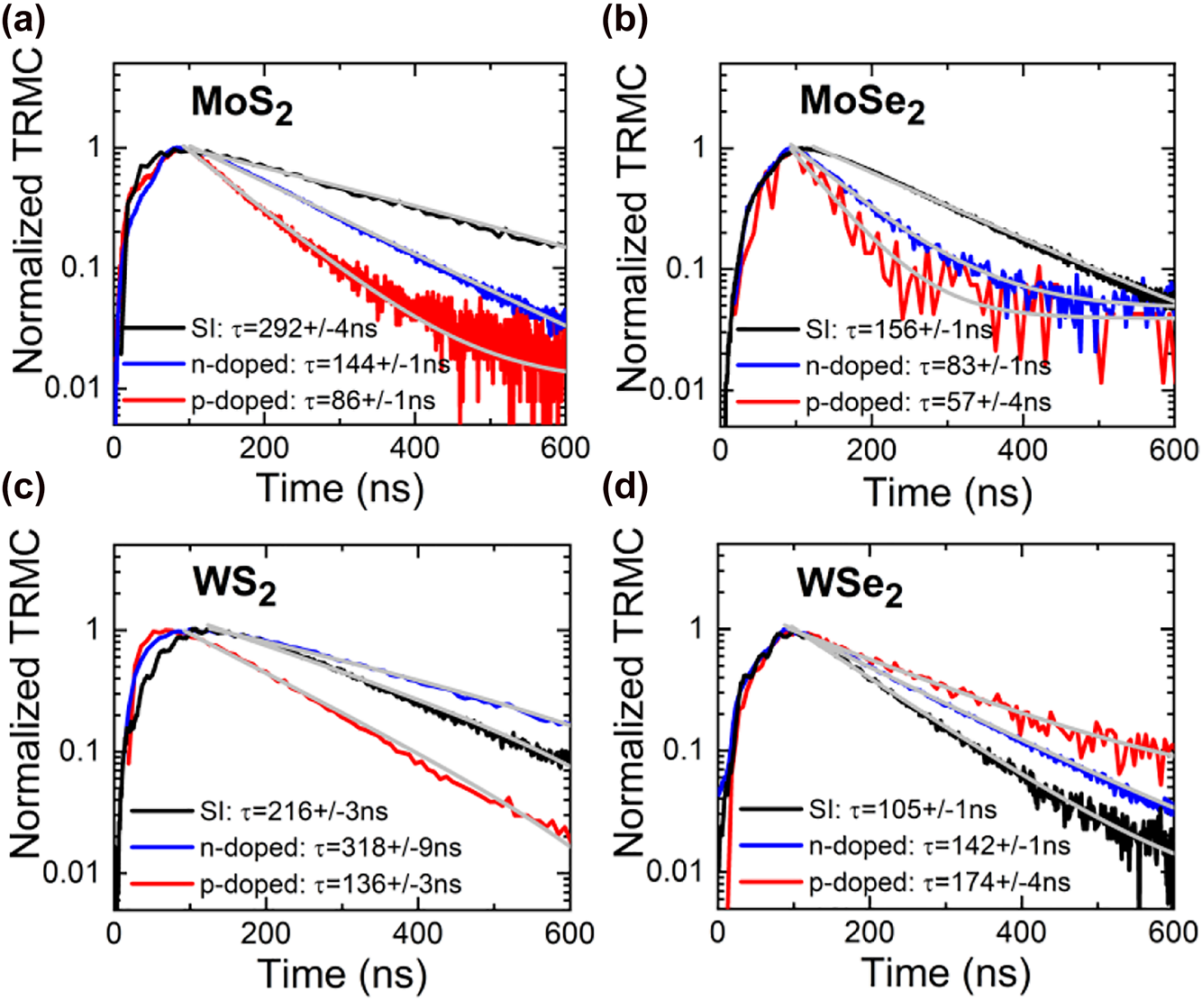
Normalized transient signal of nominally undoped (semi-insulating, SI) (black curves), *n*-doped (blue curves) and *p*-doped (red curves) TMDs obtained at room temperature. The gray lines represent the fits with single exponential decay.

In general, the concentration and activity of point defects vary from sample to sample and can be changed intentionally or unintentionally by doping. Based on that assumption we have performed TRMC experiments on bulk TMDs with doping level high enough to recognize dominant *n-* or *p-*type conductivity. It is observed that a TRMC component associated with point defects can be identified, as shown in Figure 4. Different type of conductivity of TMDs goes hand in hand with different concentration of dopants and native defects, which influence the lifetime of long-lived carriers. The lack of a clear trend for all four TMD samples after the doping is not surprising if we consider the fact that each of these materials is characterized by a different formation energy of individual defects and has different doping preferences. Without a controlled concentration of defects in TMD, we cannot conclude quantitatively about the effect of doping on the lifetime of long-lived TMDs carriers. Thus, specific defects must be identified in a given TMD and analyzed in the context of the extension (or reduction) of the TRMC signal decay time due to *n*-type (or *p*-type) doping. This is a separate research topic, and its importance calls for systematic investigation on appropriate set of TMDs, for which the defect concentration can be determined by deep level transient spectroscopy or similar techniques. Nevertheless, the measurements obtained in this work allow us to identify which component in TRMC can be attributed to point defects and estimate that its characteristic decay time is changing in the range of tenths of a microsecond for the investigated TMDs.

**Figure 5:**
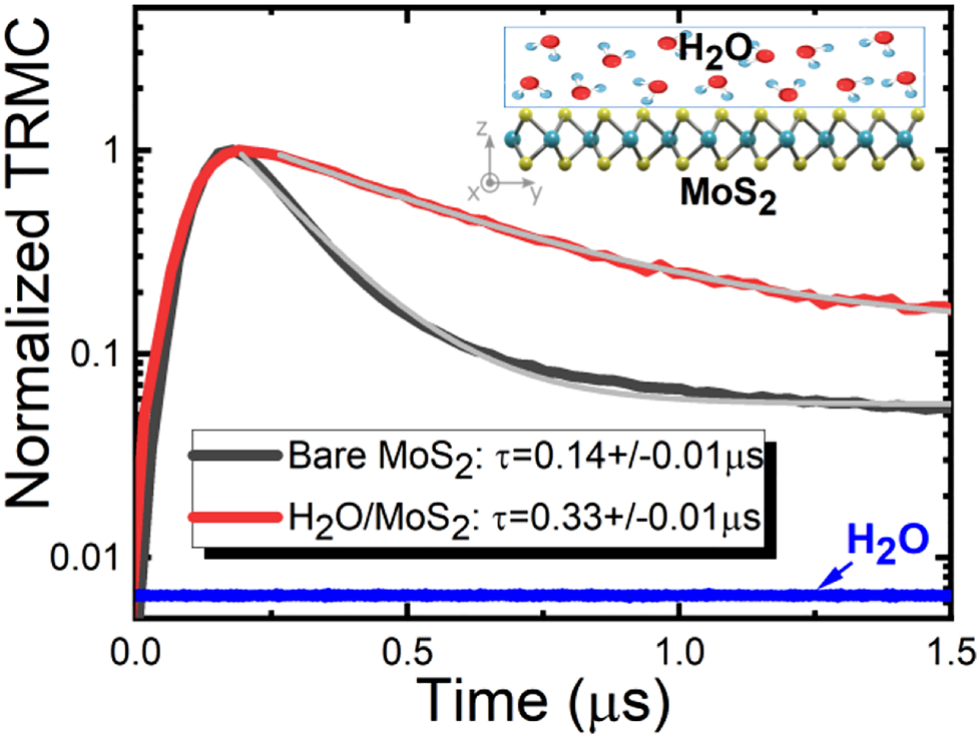
Normalized transient photoconductivity of bare MoS_2_ (black line) and MoS_2_ covered by water (red line). The gray lines represent the fits with exponential decay. No TRMC signal was observed from the water film deposited on quartz. The water TRMC trace is plotted with a blue line with an offset added to visualize it on the adopted scale in the figure.

In the case of *n*-doped or *p*-doped bulk compounds the surface band bending is an additional factor which can significantly increase the carrier lifetime due to spatial separation of carriers [52]. In the studied materials a change in band bending can be achieved by surface passivation with diverse liquids. Figure 5 shows an example of this phenomenon for MoS_2_ covered by/a thin film of deionized water where the decay time of microwave conductivity signal increases significantly. Unfortunately, such covering/passivation surface in MoS_2_ simultaneously alters the lifetime of carriers occupying surface states, making the quantitative analysis of this phenomenon difficult. However, this example clearly proves that the observed TRMC signal can be attributed to long-lived carriers, which are present in the investigated compound due to surface states, defect states, and the surface band bending. These three components determine the dynamics of TRMC signal. In this work, three components are identified by appropriate sample selection: (i) monolayer vs. bulk TMD for the analysis of surface vs. point defects in volume as the surface/volume ratio conditions are very different for such samples; (ii) TMD crystals with different point defect concentrations (nominally undoped, *n*-doped, *p*-doped) to demonstrate the sensitivity of TRMC to point defects and to define the timescale for these processes; (iii) the same TMD crystal with the modified surface to illustrate the effect of band bending (passivation of surface states) on the dynamics on TRMC signal. Quantitative studies of each of these phenomena are possible for a properly prepared series of samples and will certainly be very interesting, but they are beyond the scope of this work. Here, we selected four crystals in order to demonstrate that the observation of long-lived carriers in TMDs is a general phenomenon in these materials. We have no doubt that each of TMDs is interesting for a deeper quantitative analysis supported by additional measurements of carrier dynamics by other methods including pump-probe techniques or TRPL. However, conclusions regarding the mechanisms leading to long-lived carriers in TMDs will be the same and valid in further research.

**Figure 6:**
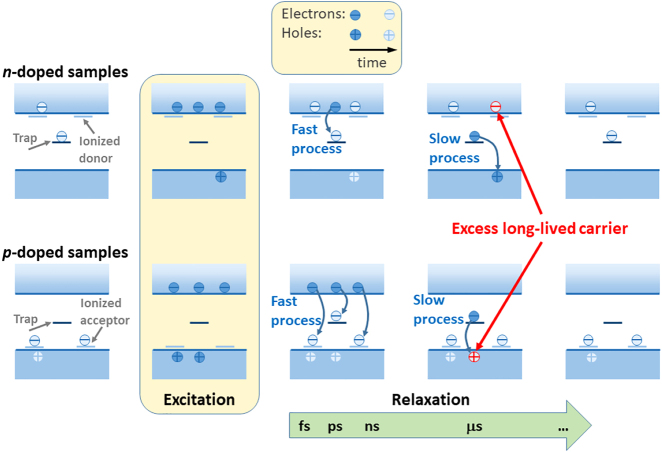
Occupation of bulk-like traps in *n*-doped and *p*-doped TMDs before, during, and after excitation with a laser pulse. Free electrons (excitons) are captured by traps within ns. All the radiative recombination (not shown in this sketch) takes place by then. The capture of electrons by traps is a fast process, as opposed to the relaxation of electrons from the traps to the valence band which is a slow process. Due to the occupation of traps by electrons, we have excess electrons in the *n*-doped sample and excess holes in the *p*-doped sample (red electrons and holes). These are long-lived carriers that are detected in time-resolved microwave conductivity.

Since the TRMC signal is observed for both *n*-doped and *p*-doped TMD samples, it is interesting to consider which type of carriers is observed in these samples. A brief consideration of this problem is illustrated in Figure 6. The sample is *n*-doped (*p*-doped) when the concentration of traps is lower than the concentration of shallow donors (acceptors). These traps can be bulk-like defect states and/or surface states. At the photoexcitation of the sample with photon energy higher than the band gap electron–hole pairs are created and electrons from traps (*n*-doped sample) and shallow acceptors (*p*-doped sample) are transferred to the conduction band. The carrier relaxation process in TMDs is very fast, as previously discussed in Figure 3: A few ns after the excitation the system is relaxed regarding the carrier thermalisation, exciton formation, as well as the radiative recombination of excitons. Since the laser pulse duration in the TRMC experiment was 3–5 ns, these relaxation processes were not observed. The process of carrier capture by traps is fast but the occupation of traps by carriers is long; that is typical for surface states and traps related to point defects. For this reason, in such compounds there is an excess of long-lived carriers, which are probed by TRMC, and these are electrons for *n*-doped and holes for *p*-doped samples, as shown in Figure 6. In general, real semiconductors can include both donors and acceptors with different activation energies and the diagram corresponding to such a situation is more complex than that shown in Figure 6. However, the conclusions regarding the type of carriers tested in TRMC remain the same: the long-lived carriers are electrons and holes in *n*-doped and *p*-doped samples, respectively.

The rise time of TRMC signal in our measurements is in the range of 0.1–0.2 μs, see Figures 4 and 5. To explain the rise time of the TRMC signal, we have to assume that capturing of excitons by traps is fast, that is expected and consistent with previous studies [13], [14], [15], [16], [17], [18], [19], [20], [21], [22], [23], [24], [25], [26], [27], [28], [29], [30], [31], [32], [33], [34, 42, 43, 56], but their dissociation is quite long and corresponds to the rise time of TRMC signal. In other words, the increase in the number of free carriers is responsible for temporal signal intensity increase immediately after the excitation pulse. Therefore, one can hypothesize that the rise time of the TRMC signal can be related to the time needed to dissociate the excitons captured by the traps. The long rise time of the TRMC signal is a strong argument that the photo-carrier relaxation process includes a rapid exciton formation and relaxation path, as shown at the top of the sketch in Figure 3.

In general, the exciton dissociation time (i.e., in this case, the time needed to break down the exciton captured by the trap) is a process that depends on the nature of the traps, temperature, and other factors, but can be longer than the process of exciton capture by traps. We expect that the part of the excitons that dissociates in this way is a small fraction of the total population of photogenerated excitons and depends on the concentration of specific traps that are responsible for this. However, this population is high enough to observe photoinduced conductivity with a characteristic long time in our experiment. Moreover, this does not exclude the possibility that photoconductivity with fast carrier dynamics may occur in the investigated material system. Such conductivity has been observed in time-resolved THz spectroscopy [17, 23, 42, 43] but cannot be detected by TRMC at the current time resolution of this method. For this reason, the rise time of the TRMC signal is interesting to study, but requires further development of the TRMC method, i.e. the use of a laser with a shorter pulse and a faster oscilloscope. In our analysis, the rise time of the TRMC signal is not performed quantitatively (fitted with theoretical curve etc.), but it has been observed that it varies between samples and therefore is assigned to the sample rather than to the system response.

The relaxation process of photogenerated carriers in TMDs also involves trapping free carriers (the bottom of the sketch shown in Figure 3), but the process is very fast and cannot be detected in our TRMC measurements due to the detection limit of ∼10 ns. It is also interesting to note that the long-lived free carriers are present in the investigated samples due to occupation of traps by carriers for a long time. This means that the lifetime of free carriers is determined by occupation time of traps by carriers.

Further exploration of long-lived carriers in van der Waals crystals by TRMC seems to be very prospective for both monolayers and heterostructures. In this work such carriers have been detected for the first time for both monolayers and bulk TMDs. This is evidence of the defect assisted dissociation of excitons into free carriers necessary for the transport of charges in solar cells or other (opto)electronic devices.

## 3 Experimental

### 3.1 Materials and methods

Monolayer and bulk TMD samples were obtained, respectively, from 2D Semiconductors (USA) and hq graphene (the Netherlands) companies. Monolayer TMDs were grown by molecular beam epitaxy on sapphire substrate [75]. Bulk TMD were grown by chemical vapor transport. *n*-doped and *p*-doped samples were doped by Au and Nb, respectively. *n*-type conductivity has been clearly confirmed for all four crystals (MoS_2_, MoSe_2_, WS_2_, WSe_2_) while *p*-type conductivity only for MoSe_2_ and WSe_2_ samples. Since the fabrication of electrical contacts to many of these materials is not well established and electrical properties are not the subject of this work, samples are named as *n*-doped and *p*-doped without electrical details, even though their electrical properties vary widely within one material (sample to sample) and also between materials.

#### 3.1.1 Raman and photoluminescence measurements

Micro-Raman and photoluminescence measurements were taken in the backscattering configuration employing a 532 nm diode-pumped solid-state laser for excitation (power 500 µW) and a 50×, NA = 0.55 microscope objective for collecting the scattered light and emission from the studied materials. A spectrometer consisting of a 0.55 m focal length grating monochromator and a liquid-nitrogen cooled Si CCD array detector was used to analyze the spectra.

#### 3.1.2 TRMC measurements

Samples were placed at the open end of a waveguide. A Gunn diode oscillator (SOL-38315-28-G1, SAGE Millimeter, Inc.) was used to generate 38.3 GHz microwaves that were directed to the waveguide. The reflected microwave power was detected using Schottky diode fine-line detector. The 532 nm line of a Continuum Minilite™ II Nd:YAG laser (frequency: 10 Hz, pulse width: 3–5 ns) was used for photogeneration of free charge carriers. Further details regarding TRMC measurements can be found in electronic Supplementary information.

## 4 Conclusions

TRMC has been applied for the first time to study carrier dynamics in both atomically thin and bulk MoS_2_, MoSe_2_, WS_2_, and WSe_2_ crystals. It has been concluded that the process of carrier (exciton) capture by traps after photoexcitation is fast, but the occupation of traps by carriers is long, which is typical of surface states and point defects. Consequently, there is an excess of long-lived carriers that is detected in TRMC. Their lifetime is close to μs and can stretch to several μs due to different contribution of surface and defect states as well as surface band bending in the case of bulk samples. The three components determine the dynamics of the TRMC signal as confirmed by TRMC study of monolayer and bulk TMDs as well as bulk TMDs with various doping (different level of native point defects) and different band bending (pristine and H_2_O-covered MoS_2_ sample). Moreover, it was concluded that the long-lived carriers are electrons and holes in *n*-doped and *p*-doped samples, respectively. We believe that the detection of long-lived carriers by TRMC is a significant step in understanding the dissociation of excitons into free-charge carriers needed for applications of TMDs in photochemistry as well as in charge transport devices including solar cells. This technique, combined with time-resolved photoluminescence, opens up new perspectives in the study of carrier dynamics in excitonic van der Waals crystals, including exciton dissociation *via* defect states. The dissociation time of excitons captured by traps has been estimated to be in the range of 0.1–0.2 μs for the studied samples. The further development of TRMC in terms of being able to measure shorter rise times is a prospect for better understanding of the exciton dissociation dynamics in TMDs. When combined with TRPL, it is an outlook for comprehensive studies of the carrier dynamics in excitonic semiconductors, including remaining van der Waals crystals, two-dimensional perovskites, and other emerging materials.

## Supplementary Material

Supplementary Material
