# Supplementary material for: Probing the long-lived photo-generated charge carriers in transition metal dichalcogenides by time-resolved microwave photoconductivity
Source: Nanophotonics. 2022 Feb 24;11(7):1335–44. doi: 10.1515/nanoph-2021-0741 (PMC11501749; doi:10.1515/nanoph-2021-0741)
Supplement: Supplementary file 1 — Supplementary Material [file j_nanoph-2021-0741_suppl.doc]

Artur P. Herman*, Szymon J. Zelewski, Kamil Misztal and Robert Kudrawiec

Origin of long-lived photo-generated charge carriers in transition metal dichalcogenides

Wroclaw University of Science and Technology, Department of Semiconductor Materials Engineering, Wybrzeże Wyspiańskiego 27, 50-370 Wrocław, Poland,
E-mail: artur.herman@pwr.edu.pl

Supporting Information

1. Time resolved microwave photoconductivity – further details

The best irf for our system comes from the length of the laser pulse and is estimated to be 10 ns. The irf deteriorates with the timebase setting on the oscilloscope (directly affecting the sample rate, equivalent to temporal resolution) and for the time window used in most measurements is estimated to be < 0.1 μs. The experimental setup for TRMC measurements is shown in **Figure S1**.


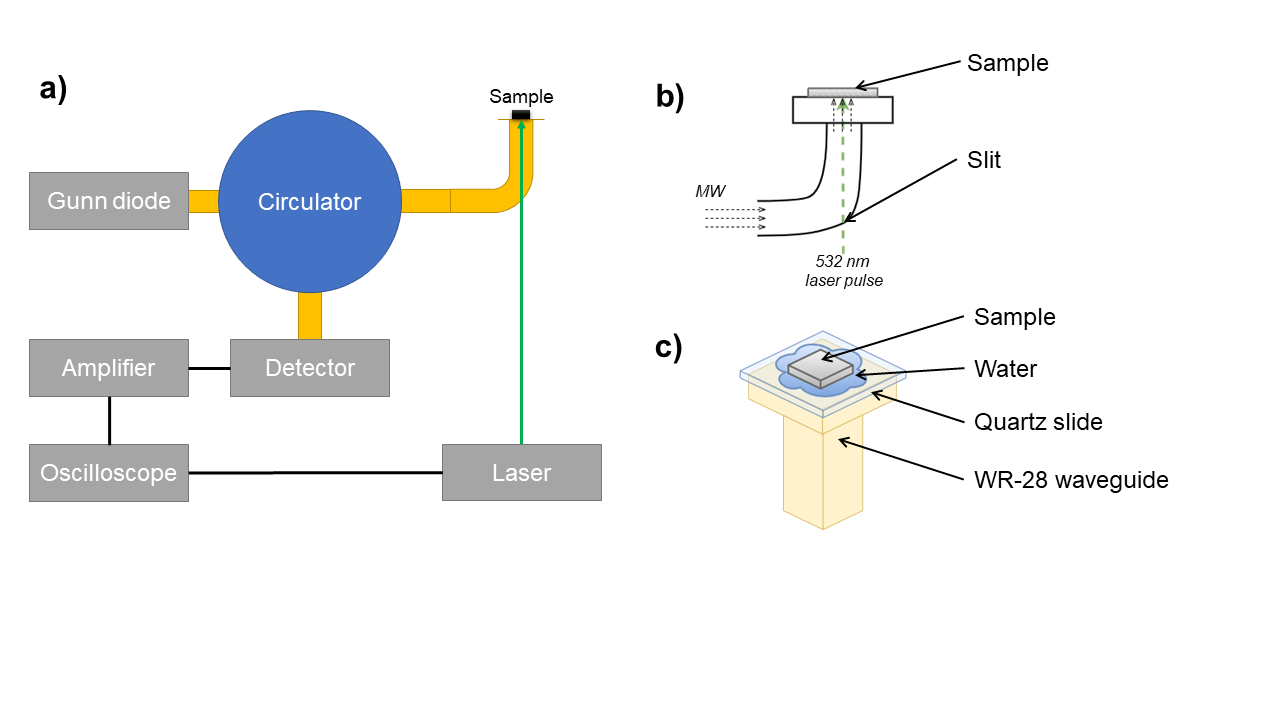


**Figure S1.** a) Schematic representation of TRMC system (resistors, attenuator, waveguide terminator, lenses and mirrors omitted for clarity) b) cross section of waveguide-end c) water contact experiment.

In this arrangement, the waveguide is directed vertically upward and the sample is placed on the open end of the waveguide, which is closed by an additional end with a position controlled by a micrometer screw. As the samples are smaller than the waveguide cross-section, they are placed on a thin quartz slide (the slide itself does not generate a signal in TRMC measurements). In the case of measurements of TMDs covered with a water film, the measurements were made for the slide/MoS_2_/water system and the slide/water as reference (**figure S1c**). Deionized water was used for these measurements and the thickness of the water film was about 0.1 mm. To check how the intensity of excitation affects the TRMC traces and the lifetime of the carriers extracted from these traces, measurements were performed increasing the excitation power by an order of magnitude from 0.46 to 4.5 mJ/cm^2^ (**figure S2**)

**a)**

**b)**

**Figure S2**. **a)** TRMC transients for MoS_2_ obtained for increasing excitation power **b)** sub-linear dependence of signal amplitude on excitation power

For MoS_2_ no significant changes in the shape of the traces were observed (**figure S2 a)**), but from the fit of curves by a sum of two exponential decays little changes in the lifetime were obtained. The *τ_1_* and *τ_2_* decreases from 0.28 to 0.23 μs (by 18%) and from 4.6 to 4.3 μs (by 7%), respectively. A similar relative decrease in carrier lifetime was observed for remaining TMD samples. The signal amplitude increases sub-linearly with the excitation power (**figure S2 b)**), which additionally means that the defects are responsible for the dynamics of the TRMC signal. It is also worth noting that even at high excitation density we deal with very low concentration of carries in this time regime, which is considered in our work, because of very fast relaxation process (i.e., fast carrier thermalization and radiative/non-radiative recombination; these processes are shorter than the duration of the laser pulse). TRMC measurements presented and discussed in this work were obtained with the excitation density of ~0.5 mJ/cm^2^. With our laser spot size, this power density gives a photon fluence of ~1.3×10^15^ cm^-2^.
